# The roles of doctors, nurses, and industrial hygienists in the healthcare management services in Korea: a comparison of the opinions of specialized health management institutions and entrusted enterprises

**DOI:** 10.1186/s40557-018-0261-9

**Published:** 2018-08-07

**Authors:** Bo-Young Jang, Dong-Mug Kang, Young-Ki Kim, Se-Young Kim, Kyung-Sun Ko

**Affiliations:** 1 Department of Occupational & Environmental Medicine, Pusan National University Yangsan Hospital, 50612 20, Geumo-ro, Mulgeum-eup, Yangsan-si, Gyeongsangnam-do Republic of Korea; 2 Department of Preventive and Occupational Medicine, School of Medicine, Pusan National University, Busan, South Korea; 3 Department of Occupational & Environmental Medicine, Pusan National University Yangsan Hospital, 20, Geumo0ro, Mulgeum-eup, Yangsan-si, Gyeongsangnam-do 626-770 South Korea; 4 Occupational Safety & Health Research Institute, Korea Occupational Safety & Health Agency, 44429 400, Jongga-ro, Jung-gu, Ulsan, Republic of Korea

**Keywords:** Health management, Specialized health management institution, Workplace, Entrustment, Performance of duties

## Abstract

**Background:**

This study aimed to identify the difference of perception about the role of appointing health officers by comparing and analyzing the response of entrustment workplace (EW) and specialized health management institution (SI). This is considered an important aspect of an institutional assessment to improve the quality of health management services.

**Methods:**

A survey questionnaire was mailed to 122 SIs and 319 EWs nationwide. The questionnaire survey was about the general characteristics of SIs and EWs and main occupations for each evaluation item. In total, 81 SIs (66.4%) and 30 EWs responded to the questionnaire. A logistic regression analysis was performed to compare the opinions of SI and EW.

**Results:**

Based on the analysis, the items showing statistically significant differences were as follows. Doctors’ main tasks survey: “Guidance on their wearing personal protective equipment (PPE)” (OR: 4.58), “Guidance of improvement of work environment (WE)” (OR: 3.33), etc.; Nurses’ main tasks survey: “Guidance on their wearing PPE” (OR: 3.86), “Guidance for programs on health process in confined space (CS)” (OR: 0.36), “Guidance on the hearing conservation program (HCP)” (OR: 0.28), etc.; Industrial hygienist (IH)‘s main tasks survey: “Guidance of work through inspection (WTI)” (OR: 0.15), “Guidance on the improvement of WE” (OR: 0.32), “Management confirmation of substances used by process and Material Safety Data Sheet (MSDS)” (OR: 0.08), “Guidance on posting or keeping of MSDS and warning signs” (OR: 0.03), “Prevention of dust-induced medical problems” (OR: 0.28), “Guidance for programs of health process in CS” (OR: 0.39), etc.

**Conclusions:**

It is necessary to educate the EWs to recognize the need for physicians to perform tasks, such as wearing a PPE, and instruction to improve WE. As for nurses’ tasks, such as education about the CS and the noise work, educating the nurses of the SI is regarded necessary as the demand of the EWs is considered. With respect to the unique tasks of IH, such as WE management and instructions for wearing PPE, among several other tasks of IH, training should be provided for improved IH recognition.

## Background

In principle, the workplace health management system of Korea requires appointing a health officers in a workplace with 300 or more employees, and health management at enterprises with 50 or more and less than 300 employees are entrusted to a specialized health management institution (SI) according to Article 16(2) of the Occupational Safety and Health Act (OSHA). However, according to Act on special measures for the deregulation of corporate activities, safety and health officers are entrusted to the management agency regardless of the size of the workplace. Therefore, the employer of workplaces with more than 300 employees also entrusts the relevant work to the specialized health management institution. When comparing the criteria for appointing health officers in other countries, Germany and France appoint occupational health doctors regardless of the industry type and size, and in the case of Korea and Japan, enterprises with 50 or more employees health officers [1]. In Korea, SIs employ doctors, nurses, and industrial hygienists (IHs) who regularly visit enterprises to manage the work environment (WE) of the enterprises and provide health management and health education to workers. The roles of appointing health officers are divided into 14 categories and specified in the OSHA. As of March 2016, enterprises notified of the appointment of enterprise health administrators were 19,932. Of these, 4406 employed appointing health officers, and 15,526 (77.9%) healthcare-entrusted services (HES) [2]. As of 2016, total number of enterprises in Korea is 2,457,225, and the total number of workers is 18,431,716. Of these, 94% of all industrial accidents occur in enterprises with less than 300 employees, and specifically, 96% of work accidents, 82% of deaths, and 68% of occupational diseases occur in enterprises with less than 300 employees. According to the status of industrial accidents at enterprises that carry out health management through the services of SI, 12.7% of all industrial accidents, 50% of all accident on duty, 22% of all deaths, 23% of all occupational diseases take place in enterprises with 50–299 employees [3]. According to the results of the health examination (HE) of workers in 2016, the proportions of suspected general diseases (C2), diagnosed general diseases (D2), suspected occupational diseases (C1), and diagnosed occupational diseases (D1) were 37.0, 34.9, 36.1, and 28.7%, respectively [4]. In a study on the participation rate of general HE of workers by the size of the business based on the data from the National Health Insurance Corporation from 2006 to 2013, it was found that the participation rate of enterprises with less than 50 non-white-collar workers and 50–299 non-white-collar workers were lower than that of enterprises with more than 300 non-white-collar workers [5]. Therefore, both health management of EW and quality management of these services are deemed important.

In the case of HES, standardization is thought to be required since various occupations provide HES. The guidelines for standardization of the SI were developed by analyzing the types and proportion of work performed by each occupation in relation to the roles of appointing health officers prescribed by the OSHA of 2016. It is expected that the evaluation items of SI will be improved and implemented. Previously, there have been some studies on HES. In 1997, a study examined the evaluation of HES, wherein HES were assessed in terms of workforce and geographic accessibility, persistence and inclusiveness of services, quality of service, provision of preventive services, and participation of workers and employers [6]. However, the study did not evaluate doctor’s work, and only 50% of the target institutions had participated. According to a study on the health management status of enterprises conducted by doctors, nurses and IHs of SI, 41.5% of the respondents provided follow-up care for workers who diagnosed diseases. Among the health management items, general health counseling and lifestyle counseling were relatively high, and health promotion activities were relatively low in the workplace [7]. This study has limitations in that it does not reflect the opinion of the workplace because only analyzed the questionnaires of each occupation of SI. In another study on the perception and attitude of the health management entrustment service of 149 small- and medium-sized enterprises in the Incheon area, the perception of related laws, health management interest, and health management task perception were lower than average. The degree of awareness and interest in occupational diseases showed above average awareness. Unlike our study, the responses were examined by the health officer at the workplace, and no investigation was conducted on the healthcare professional personnel [8]. According to a survey of workers’ need and willingness of SI to health management services in the workplace, 81% of the workers required medication and care at the workplace, and more than 80% of doctors and nurses responded that they need medication and care in the workplace. This indicates that the healthcare entrustment services are highly demanded by workers, and their doctors and nurses recognized them as major tasks [9]. Unlike our study, this study conducted an opinion survey on both SI and entrustment workplace (EW). However, this survey was limited to in-work medical examination, and no investigation was conducted on the IH of the SI.

The existing studies have been conducted only on the SI [7] or the EW [8], and only a few studies have compared the two [9]. Some studies have compared the opinions of SIs and EWs but there are limitations in that the results on specific items, such as the medical service in the workplace, were not specific and standardized. The evaluation items of the SIs to be implemented in the future seek to improve the quality of the HES and define the role of the appointing health officers through the standardization of the work of health officials. Therefore, prior to the evaluation of the healthcare professionals, it is necessary to conduct an opinion survey on both the SI and the EW regarding the duties and roles of the appointing health officers based on specific criteria. Therefore, the aim of this study was to identify the differences in the perceptions toward workplace appointing health officer’s role and help develop HES by comparing and analyzing the responses of EWs and each occupations of the SIs about the evaluation item for SIs.

## Methods

### Survey on the status of specialized health management institution

The survey was conducted with a total of 122 SIs nationwide in two stages from July 24, 2017 to September 15, 2017.

The survey was mailed and emailed, and responses were made by one of the administrators, doctors, nurses, and IHs of the relevant institution via mail, e-mail, or fax. Eighty-one of the 122 institutions (66.4%) responded to the survey. The contents of the survey included the status and general characteristics of the organization’s workforce, the appropriateness to the institution’s evaluation items, and occupation. The questionnaire was administered to the proper occupation for each major duty in the item “Fidelity of technical guidance” among the institutional evaluation items of the SI, and the responses were scored as “yes” or “no.” We surveyed the qualifications of each occupation (doctors, nurses, IH qualifications) and the number of business establishments that provided HES, industry, and inspectors. A division was made by university hospitals, clinics, and corporate organizations. The entrustment sites were divided into 50–99 employees, 100–199 employees, 200–299 employees, and 300 or more employees. Businesses in the workplace were classified into four categories (manufacturing 1, manufacturing 2, service business, and construction) by simplifying the OSHA; The kind and scale of business to which a health officer and the number and method of designation of health officer. (Appendix Table 4).

### Survey on the status of entrustment workplace

The survey was conducted in two stages from August 24, 2017 to September 15, 2017 with a total of 16 groups, 319 workplaces considering industry type (manufacturing 1, manufacturing 2, service business, construction) and size (50–99 employees, 100–199 employees, 200–299 employees, 300 or more employees).

The questionnaire was mailed, and responses were made via mail, e-mail, or fax. Thirty out of 319 workplaces (9.4%) responded. The survey included the type of industry and the number of workers, the adequacy of the institutional evaluation items, and proper occupations for each item. Workplace was divided into four types of industries and sizes as mentioned above. The manager of the workplace stated which occupation is proper for the “Improvement of technical guidance” among the institutional evaluation items for the SI. The responses were scored as “yes” or “no.”

### Analyses

The frequencies and proportions of the responses to the questionnaires were analyzed using chi-square test, and statistical significance was confirmed; a simple logistic regression analysis was performed to compare the opinions of the SI and EW.

In the analysis of the frequencies and proportions, when the answer as main task over 50%, it was defined as the recognition of the main task, and when the answer as main task below 50%, it was defined as no recognition of the main task. All analyses were performed using the SPSS Statistical Program 23.0 (SPSS Inc., Chicago, IL). Statistical significance was defined as the *p*-value less than 0.05.

## Results

### Survey on the status of specialized health management institution

#### General characteristics

We classified participating institutions by type. They were 19 university hospitals (23.5%), 42 clinics (51.9%), and 20 corporations (24.7%). In the status of the EW, the number of EWs and the number of workers in enterprises with 50–99 employees were the highest. By industry, the highest industry was manufacturing 1, followed by service business, and manufacturing 2 (Appendix Table 5).

#### Occupation in charge of major tasks of the tasks and roles of health officer

Responses to the tasks listed in the item “Improvement of technical guidance” among the evaluation items of the SI are shown in Table 1. Among these, all items except for “Guidance of WTI” and “Management of executory places of business” were statistically significant.

**Table 1:** Responses of individuals of each occupation to items considered to be their tasks (specialized health management institution)

Items	Detailed items	Doctor (n(%))	Nurse (n(%))	IH^†^ (n(%))	Χ^2††^	p-value
1.1 Guidance on work place health management						
	1.1.1 The assessment of work fitness of workers with health problems identified by the HE^‡^	53(70.7)	23(29.1)	5(6.3)	72.6	0.000
	1.1.2 Follow-up of workers’ HE	55(73.3)	68(86.1)	5(6.3)	116.8	0.000
	1.1.3 Investigation of medical problems and guidance on the prevention of their recurrence	44(58.7)	34(43.0)	14(17.5)	28.2	0.000
	1.1.4 Participation rate of occupational health and safety committee	10(13.3)	26(32.9)	24(30.0)	8.9	0.013
	1.1.5 Guidance on wearing PPE^§^	31(41.3)	59(74.7)	45(56.3)	17.6	0.000
	1.1.6 Guidance on the improvement of WE^∥^	30(40.0)	23(29.1)	39(48.8)	6.4	0.039
	1.1.7 Guidance on WTI^¶^	37(49.3)	45(57.0)	45(56.3)	1.1	0.523
	1.1.8 Guidance on the formulation of plans for health education and the conduction	27(36.0)	60(75.9)	28(35.0)	34.3	0.000
1.2 Performance of prevention of occupational diseases						
	1.2.1 Management confirmation of substances used by process and MSDS	17(22.7)	18(22.8)	41(51.3)	19.5	0.000
	1.2.2 Guidance on posting or keeping MSDS and warning signs	10(13.3)	31(39.2)	40(50.0)	21.1	0.009
	1.2.3 Guidance on investigation of risk factors of musculoskeletal disorders	36(48.0)	24(30.4)	43(53.8)	9.5	0.009
	1.2.4 Guidance on campaigns to improve workers’ health	37(49.3)	61(77.2)	13(16.3)	59.4	0.000
	1.2.5 Guidance on the prevention of job stress	42(56.0)	52(65.8)	11(13.8)	49.1	0.000
	1.2.6 Guidance on the assessment of cerebro-cardiovascular risk	44(58.7)	55(69.6)	5(6.3)	73.7	0.000
	1.2.7 Guidance for programs on health process in CS^#^	14(18.7)	12(15.2)	41(51.3)	30.7	0.000
	1.2.8 Prevention of dust-induced medical problems	29(38.7)	19(24.1)	42(52.5)	13.6	0.001
	1.2.9 Guidance on HCP^*^	31(41.3)	21(26.6)	43(53.8)	12.2	0.002
1.3 Management of executory places of business		16(21.3)	25(31.6)	31(38.8)	5.6	0.070
1.4 Rate of accreditation of risk assessment		7(9.3)	0(0.0)	21(26.3)	26.7	0.000

† IH: industrial hygienist, ‡ HE: Health examination, § PPE: Personal protective equipment, ∥ WE: Work environment, ¶ WTI: Work through inspection, # CS: Confined space, * HCP: Hearing conservation program †† Chi-square test

##### *Doctors*

The items that the doctor considered as main tasks in the workplace were “The assessment of work fitness of workers with health problems identified by the HE” (70.7%), “Follow-up of workers’ HE” (73.3%), “Investigation of medical problems and guidance on the prevention of their recurrence” (58.7%), “Guidance on the prevention of job stress” (56.0%), “Guidance on the assessment of cerebro-cardiovascular risk” (58.7%), etc. It was related to medical services based on medical knowledge. Among the items not considered to be the main tasks of the doctors, “Rate of accreditation of risk assessment” (9.3%), “Participation rate of occupational health and safety committee” (13.3%), “Guidance on posting or keeping of MSDS” (13.3%) were less considered as major tasks.

##### *Nurses*

The items considered as the main tasks of nurses in the workplace included “Follow-up of workers’ HE” (86.1%), “Guidance on wearing PPE” (74.7%), “Guidance on WTI” (57.0%), “Guidance on the formulation of plans on health education and the conduction” (75.9%), “Guidance on campaigns to improve workers’ health” (77.2%), “Guidance on prevention of job stress” (65.8%), “Guidance on assessment of cerebro-cardiovascular risk” (69.6%), etc. Among the items not considered to be the main tasks of nurses, the items “risk assessment accreditation rate” 0.0 (%), “Guidance on programs of health process on CS” (15.2%) were less considered as major tasks.

##### *Industrial hygienists*

The items considered as the main tasks of IHs in the workplace included “Guidance on wearing PPE” (56.3), “Guidance on WTI” (56.3%), “Management confirmation of substances used by process and MSDS” (51.3%), “Guidance on posting or keeping of MSDS and warning signs” (50.0%), “Guidance on the investigation of risk factors of musculoskeletal disorders” (53.8%), “Guidance for the programs on health process in CS” (51.3%), “Prevention of dust-induced medical problems” (52.5%), “Guidance on HCP” (53.8%), etc. These tasks were related to the improvement of and guidance related to WE. Among the items not considered to be the main tasks of IHs, “The assessment of work fitness of workers with health problems identified by the HE” (6.3%), the items “Follow-up of workers’ HE” (6.3%), “Guidance on the assessment of cerebro-cardiovascular risk” (6.3%) were less considered as major tasks. These were related to medical services based on medical knowledge.

In the survey on IH, compared to other occupations, the responses were not as high as expected, including items that were considered to be unique tasks, as their main job. Therefore, an additional analysis was conducted to identify the differences in the responses according to the institutional type and the qualification of IH in participating institutions. No statistically significant results were found (data not shown).

### Survey on the status of entrustment workplace

#### General characteristics

Thirty of the 319 enterprises (9.4%) responded to the survey. By industry, the highest industry was service business (36.7%), followed by manufacturing 1 (23.3%) and manufacturing 2 (23.3%), and construction (16.7%). By enterprise size, the number of enterprises with 200–299 employees was the highest (33.3%) (Appendix Table 6).

#### Occupation in charge of major tasks of the tasks and roles of health officer

In the evaluation items of the SI, the managers of the workplace responded which occupations were the most responsible for the tasks listed in the item “Improvement of technical guidance.” Among these, all the items were statistically significant (Table 2).

**Table 2:** Responses of entrustment workplace to items considered to be the main tasks and roles of each occupation

Items	Detailed items	Doctor (n(%))	Nurse (n(%))	IH^†^ (n(%))	Χ^2††^	p-value
1.1 Guidance on work place health management						
	1.1.1 The assessment of work fitness of workers with health problems identified by the HE^‡^	23(76.7)	19(63.3)	2(6.7)	33.2	0.000
	1.1.2 Follow-up of workers’ HE	22(73.3)	26(86.7)	0(0.0)	52.5	0.000
	1.1.3 Investigation of medical problems and guidance on the prevention of their recurrence	19(63.3)	17(56.7)	6(20.0)	13.1	0.001
	1.1.4 Participation rate of occupational health and safety committee	3(10.0)	20(66.7)	10(33.3)	21.0	0.000
	1.1.5 Guidance on wearing PPE^§^	4(13.3)	13(43.3)	23(76.7)	24.4	0.000
	1.1.6 Guidance on the improvement of WE^∥^	5(16.7)	8(26.7)	26(86.7)	35.0	0.000
	1.1.7 Guidance on WTI^¶^	4(13.3)	14(46.7)	24(80.0)	26.8	0.000
	1.1.8 Guidance on the formulation of plans for health education and the conduction	8(26.7)	18(60.0)	20(66.7)	11.0	0.004
1.2 Performance of prevention of occupational diseases						
	1.2.1 Management confirmation of substances used by process and MSDS	2(6.7)	7(23.3)	28(93.3)	52.4	0.000
	1.2.2 Guidance on posting or keeping MSDS and warning signs	1(3.3)	8(26.7)	29(96.7)	58.0	0.000
	1.2.3 Guidance on investigation of risk factors of musculoskeletal disorders	11(36.7)	20(66.7)	19(63.3)	6.6	0.037
	1.2.4 Guidance on campaigns to improve workers’ health	11(36.7)	23(76.7)	9(30.0)	15.3	0.000
	1.2.5 Guidance on the prevention of job stress	14(46.7)	24(80.0)	7(23.3)	19.5	0.000
	1.2.6 Guidance on the assessment of cerebro-cardiovascular risk	19(63.3)	20(66.7)	4(13.3)	21.5	0.000
	1.2.7 Guidance for programs on health process in CS^#^	1(3.3)	10(33.3)	22(73.3)	31.9	0.000
	1.2.8 Prevention of dust-induced medical problems	2(6.7)	10(33.3)	24(80.0)	34.4	0.000
	1.2.9 Guidance on HCP^*^	5(16.7)	17(56.7)	19(63.3)	15.4	0.000
1.3 Management of executory places of business		5(16.7)	14(46.7)	19(63.3)	13.8	0.001
1.4 Rate of accreditation of risk assessment		2(6.7)	5(16.7)	22(73.3)	35.5	0.000

† IH: industrial hygienist, ‡ HE: Health examination, § PPE: Personal protective equipment, ∥ WE: Work environment, ¶ WTI: Work through inspection, # CS: Confined space, * HCP: Hearing conservation program †† Chi-square test

##### *Doctors*

The items considered to be the main tasks of the doctors in the workplace were “the assessment of work fitness of workers with health problems identified by the HE” (76.7%), “Follow-up of workers’ HE” (73.3%), “Investigation of medical problems and guidance on the prevention of their recurrence” (63.3%), “Guidance on the assessment of cerebro-cardiovascular risk” (63.3%), etc. Among the items not considered to be the main tasks of the doctors, “Management confirmation of substances used by process and MSDS” (6.7%), “Guidance on posting or keeping of MSDS and warning signs” (3.3%), “Guidance for programs on health process in CS” (3.3%), “Prevention of dust-induced medical problems” (6.7%) items were less considered as major tasks.

##### *Nurses*

The items that the nurses considered to be the main charge in the workplace were ‘The assessment of work fitness of workers with health problems identified by the HE’ (63.3%), ‘Follow up of workers’ HE’ (86.7%), ‘Investigation of medical problems and guidance on the prevention of their recurrence’ (56.7%), ‘Participation rate of occupational health and safety committee ‘(66.7%), ‘Guidance on the formulation of plans on health education and the conduction’ (60.0%), ‘Guidance of investigation of risk factors of musculoskeletal disorders’ (66.7%), ‘Guidance of campaigns to improve workers’ health’ (76.7%), ‘Guidance of HCP’ (56.7%) etc. Among the items not considered to be the main tasks of the nurses, ‘Rate of accreditation of risk assessment’ items were less considered as major tasks.

##### *Industrial hygienists*

The items that the IHs considered to be the main tasks in the workplace included “Guidance on wearing PPE” (76.7%), “Guidance on the improvement of working environment’ (86.7%), “Guidance on WTI” (80.0%), “Guidance on the formulation of plans on health education and the conduction” (66.7%), “Management confirmation of substances used by process and MSDS” (93.3%), “Guidance on posting or keeping of MSDS and warning signs” (96.7%), “Guidance on the investigation of risk factors of musculoskeletal disorders” (63.3%), “Guidance for programs on health process in CS” (73.3%), “Prevention of dust-induced medical problems” (80.0%), “Guidance on HCP’ (63.3%), “Management of executory places of business” (63.3%), ‘Rate of accreditation of risk assessment” (73.3%), etc. Among the items not considered to be the main tasks of the IH, ‘The assessment of work fitness of workers with health problems identified by the HE” (6.7%), ‘Follow up of workers’ HE’ (0.0%) were less considered as major tasks.

#### Comparison of the survey on specialized health management institution and entrustment workplace

The results of the questionnaire survey on the evaluation items of the SI were compared and analyzed with those of the SI and EW. The numbers in parentheses indicate the odds ratio value for the item (Table 3).

**Table 3:** Comparison of the responses of institutions and entrustment workplace to major occupational roles in the evaluation items through simple logistic regression analysis

Items	Detailed items		Doctor’ role	Nurse’s role	IH∥‘s role
			O.R.^***^(95%CI^§§§^)	O.R. (95%CI)	O.R. (95%CI)
1.1 Guidance of work place health management					
	1.1.1 The assessment for work fitness of workers with health problems identified by the HE¶	EW^‡^	1.0(ref)	1.0(ref)	1.0(ref)
		SI^§^	0.733(0.275, 1.956)	0.238*(0.098, 0.577)	0.900(0.165, 4.916)
	1.1.2 Follow up of workers’ HE	EW	1.0(ref)	1.0(ref)	1.0(ref)
		SI	1.000(0.384, 2.605)	0.805(0.240, 2.694)	> 100(0.000, > 100)
	1.1.3 Investigation of medical problems and guidance on the prevention of their recurrence	EW	1.0(ref)	1.0(ref)	1.0(ref)
		SI	0.822(0.343, 1.968)	0.578(0.247, 1.350)	0.848(0.293, 2.460)
	1.1.4 Participation rate of occupational health and safety committee	EW	1.0(ref)	1.0(ref)	1.0(ref)
		SI	1.385(0.353, 5.427)	0.245*(0.100, 0.599)	0.857(0.349, 2.102)
	1.1.5 Guidance on their wearing of PPEs††	EW	1.0(ref)	1.0(ref)	1.0(ref)
		SI	4.580*(1.452, 14.443)	3.858*(1.596, 9.323)	0.391(0.151, 1.016)
	1.1.6 Guidance of improvement of WE#	EW	1.0(ref)	1.0(ref)	1.0(ref)
		SI	3.333*(1.148, 9.675)	1.129(0.440, 2.902)	0.146*(0.04, 0.458)
	1.1.7 Guidance of WTI‡‡	EW	1.0(ref)	1.0(ref)	1.0(ref)
		SI	6.329*(2.012, 19.906)	1.513(0.650, 3.519)	0.321*(0.119, 0.872)
	1.1.8 Guidance on the formulation of plans on health education and the conduction	EW	1.0(ref)	1.0(ref)	1.0(ref)
		SI	1.547(0.606, 3.946)	2.105(0.861, 5.149)	0.269*(0.111, 0.654)
1.2 Performance of prevention of occupational disease					
	1.2.1 Management confirmation of substances used by process and MSDS	EW	1.0(ref)	1.0(ref)	1.0(ref)
		SI	4.103(0.886, 19.008)	0.970(0.358, 2.625)	0.075*(0.017, 0.337)
	1.2.2 Guidance of posting or keeping of MSDS and warning signs	EW	1.0(ref)	1.0(ref)	1.0(ref)
		SI	4.462(0.545, 36.496)	1.776(0.703, 4.486)	0.034*(0.004, 0.265)
	1.2.3 Guidance of investigation of risk factors of musculoskeletal disorders	EW	1.0(ref)	1.0(ref)	1.0(ref)
		SI	1.594(0.668, 3.805)	0.218*(0.089, 0.535)	0.673(0.284, 1.595)
	1.2.4 Guidance of campaigns to improve workers’ health	EW	1.0(ref)	1.0(ref)	1.0(ref)
		SI	1.682(0.705, 4.013)	1.031(0.381, 2.793)	0.453(0.170, 1.208)
	1.2.5 Guidance of prevention of job stress	EW	1.0(ref)	1.0(ref)	1.0(ref)
		SI	1.455(0.622, 3.403)	0.481(0.176, 1.320)	0.524(0.182, 1.510)
	1.2.6 Guidance of risk assessment of cerebro-cardiovascular risk	EW	1.0(ref)	1.0(ref)	1.0(ref)
		SI	0.822(0.343, 1.968)	1.146(0.467, 2.812)	0.433(0.108, 1.737)
	1.2.7 Guidance of programs of health process on CS**	EW	1.0(ref)	1.0(ref)	1.0(ref)
		SI	6.656(0.835, 53.082)	0.358*(0.135, 0.951)	0.382*(0.152, 0.960)
	1.2.8 Prevention of dust induced medical problems	EW	1.0(ref)	1.0(ref)	1.0(ref)
		SI	8.826*(1.954, 39.871)	0.633(0.253, 1.586)	0.276*(0.102, 0.748)
	1.2.9 Guidance of of HCP†††	EW	1.0(ref)	1.0(ref)	1.0(ref)
		SI	3.523*(1.215, 10.214)	0.277*(0.115, 0.666)	0.673(0.284, 1.595)
1.3 Management of executory places of business		EW	1.0(ref)	1.0(ref)	1.0(ref)
		SI	1.356(0.448, 4.105)	0.529(0.224, 1.250)	0.366*(0.154, 0.873)
1.4 Rate of accreditation of risk assessment		EW	1.0(ref)	1.0(ref)	1.0(ref)
		SI	1.441(0.282, 7.370)	0.000(0.000,)	0.129(0.050, 0.335)

* p-value< 0.05, ‡ EW: Entrustment workplace, § SI: Specialized health management institution, ∥ IH: Industrial hygienist, ¶ HE: Health examination, # PPE: Personal protective equipment, †† WE: Work environment, ‡‡ WTI: Work through inspection, ** CS: Confined space, ††† HCP: Hearing conservation program, ***O.R.: Odds ratio, §§§95% CI: 95% Confidence interval

##### *Doctors*

Based on the comparison and analysis of the responses to occupations in charge of the major tasks of the doctors in the evaluation items of the SI and the EW, “Guidance on their wearing of PPE” (OR: 4.58), “Guidance on improvement of working environment” (OR: 3.33), “Guidance on WTI” (OR: 6.33), “Prevention of dust-induced medical problems” (OR: 8.83), “Guidance on HCP” (OR: 3.52) were statistically significant. For each item, the rate of EWs responding that doctors are in charge of major tasks in all items was low, and doctors were more likely to consider these as their main tasks.

##### *Nurses*

Based on the comparison and analysis of the responses to occupations in charge of the major tasks of the nurses in the evaluation items of the SI and EW, “The assessment of work fitness of workers with health problems identified by the HE” (OR: 0.24), “Participation rate of occupational health and safety committee” (OR: 0.25), “Guidance on wearing PPE” (OR: 3.86), “Guidance on the investigation of risk factors of musculoskeletal disorders” (OR: 0.22), “Guidance for programs on health process in CS” (OR: 0.36), “Guidance on HCP” (OR: 0.28) were statistically significant. The rate of EWs responding that nurses are in charge of the major tasks of all items excluding “Guidance on wearing PPE” was high; the rate of nurses responding that nurses are in charge of the major tasks in these items of the same questionnaire was low.

##### *Industrial hygienist*

Based on the comparison and analyses of the responses to occupations in charge of the major tasks of the IH in the evaluation items of the SI and EW, “Guidance on WTI” (OR: 0.15), “Guidance on the improvement of working environment” (OR: 0.32), “Guidance on the formulation of plans on health education and the conduction” (OR: 0.27), “Management confirmation of substances used by process and MSDS” (OR: 0.08), “Guidance of posting or keeping of MSDS and warning signs” (OR: 0.03), “Prevention of dust-induced medical problems” (OR: 0.28), “Management of executory places of business” (OR: 0.37), “Guidance for programs on the health process in CS” (OR: 0.39) were statistically significant. The proportion of respondents who answered that they were in charge of the main tasks of the IH for all items was higher in the EW than the IHs.

## Discussion

Through this study, we were able to identify the difference between SI and EW on the recognition of health management for variable occupations. According to the doctors, medical practice is a major role for doctors in both the physician and the workplace. However, in safety-related work, there was few answers defining it as doctor’s role. Nurses replied their major role was the follow up of workers’ HE. Though, some differences of opinion existed in terms of Guidance on wearing PPE, Guidance on investigation of risk factors of musculoskeletal disorders, Guidance for programs on health process in CS, and Guidance on HCP. In response to the IHs, the EWs are highly demanded for safety-related aspects, such as wearing PPE, managing the WE, and prevention of health problems due to work environment. But, half of the IHs answered that these items were the main tasks of the IH, such as the management of the WE and the instruction to wear PPE, that were recognized as unique aspects of ​​IH.

The results of the comparative analysis of the responses of the SIs and EW are as follows. According to the analysis of the responses to doctors’ work, both physicians and EWs were considered to be the main occupational areas in terms of medical behaviors, such as the assessment of work fitness and follow up of workers’ HE. However, in the case of safety areas, such as wearing PPE, improvement of working environment, and prevention of dust-induced medical problems, very few respondents stated that it was the doctor’s main task in EWs. The EWs regarded only the aspect of providing medical knowledge by physicians as important but the doctors recognized the improvement of WE is a major task that could affect the health of the workers. The German ISHA (Arbeitsschutzgesez) defines the duties of industry as follows. The occupational physicians should carry out occupational medical examination, supervision of actual situation of industrial safety and disaster prevention, including wearing PPE during WTI, connection with other occupations for WE problems including selection of PPE, and education of workers [10]. Guidelines on the role of the Occupational Physician of the British Medical Association suggest that the occupational physicians are obligated to monitor the health of the workers, monitor the risk of the workplace, assess the fitness of work, and make policy decisions for the welfare of the workers [11]. However, the result of this study showed that EWs do not properly perceive the doctor’s work on the improvement of WE and prevention of disasters, including the wearing and selection of PPE. It is considered that the longer visit period is reflected in these results compared to other occupations. In case of HES, the visit period for each occupation should be quarterly for doctors, monthly for nurses, and bimonthly for IHs, in case of more than 100 employees. In case of less than 100 employees, the physician is required to visit semi-annually, the nurse monthly, and the IHs quarterly. In general, the doctors did not visit the workplaces frequently that they are required to provide adequate healthcare service in such a limited time. Therefore, many of them prioritize to provide healthcare service rather than other task such as field inspection and guidance of PPE. For this reason, EW tend to limit doctor’s role as a healthcare provider due to their lack of experience in on-site health guidance.

According to the analysis of the responses to the nurses’ tasks, follow up of workers’ HE was recognized as the main tasks of the nurses by both nurses and the EW. However, in the case of providing instructions regarding PPE items, there was more response that it was a nurse’s main task in nurses(SI) than in EW. In the case of musculoskeletal hazard analysis, health process program in CS, and instruction regarding HCP items, the respondents in the EW answered that these tasks are considered the main tasks of the nurses. Among the items that were statistically significant, a higher percentage of respondents in EW compared to nurses stated that the evaluation items were nurses’ main tasks in all items except for wearing PPE. The nurses are required to visit the EW once a month in the HES. It is considered that the shorter visit period is reflected in these results compared to other occupations. According to a 2007 study on the role of industrial nurses in the workplace, nurses are expected to carry out primary prevention of injuries or illnesses of workers, emergency treatment, general treatment services, comprehensive medical diagnosis, management plans for individuals and groups and general health counseling and evaluation [12]. The results of this study are as follows. It is important for the EWs to perform primary preventive work on workers’ health as well as medical support work, which are the main health management tasks of nurses. Therefore, it is necessary to educate nurses about preventive works, such as guidance of programs of health process on CS, guidance of HCP, etc. However, considering that the EWs have answered that the major tasks of the nurses are related to many of the items of the institutional evaluation items, it is necessary to coordinate with the enterprise to ensure that the workload of the nurses does not increase excessively.

The analysis of the responses to the main tasks of IHs showed that the EWs are highly demanded for safety-related aspects, such as wearing PPE and managing the WE, and prevention of health problems due to work environment, such as education related to CS work and preventing dust-induced medical problems. However, in this study, the responses of IHs from SIs did not exceed over 60% of the respondents, who answered that they are in charge of the main tasks in most items. Further, half of the respondents answered that these items were the main tasks of the IH, such as the management of the WE and the instruction to wear PPE, that were recognized as unique aspects of ​​IH. According to Workplace Health Management Guidelines of Institution of Safety and Health and the International Institute of Industrial Hygiene, IH must monitor workplace exposure through risk assessments and monitor occupational health hazards, such as noise, dust, and chemicals and instructions for wearing PPE [13, 14]. In 1994, Kim et al. performed a job analysis of health officers in the health entrustment service. They analyzed the responses from 40 nurses, 11 IHs, and 6 physicians from 15 SIs. Both nurses and physicians recognized occupational and general disease susceptibility management and development of health education materials as priorities. In the case of occupational hygiene workers, tasks, such as measuring and evaluating WE and improving working environment, were stated as the priority tasks. Both nurses and doctors recognized the priority task of managing occupational and general illnesses and development of health education programs. In the case of IHs, tasks, such as measuring and evaluating work environment and improving WE were stated as priority tasks [15]. Similar to previous studies, doctors and nurses in our study perceived follow-up health management and general counseling as the main tasks. In the case of IHs, the percentage of respondents who answered that they were in charge of the main tasks of the safety areas, such as the instruction for wearing PPE and WE management, was not high compared to the previous studies. To analyze the characteristics of the responses of the IH in this study by the general characteristics of the SI, an additional analysis was performed. There were no statistically significant differences between the results of the analysis of responses by institutional type and IH qualification. Therefore, additional research is needed to examine the adequacy of perceptions of major tasks and the causes of perceptions of IHs.

This study has some limitations. First, the proportion of EWs responding to the survey was low. Thirty of the 319 enterprises responded to the questionnaire. Considering the categories of industry, size, and area, 160 enterprises in 16 groups were first sent by post with the cooperation letter of the Ministry of Employment and Labor. In the first survey, the response rate was low, at 6.3%. Then, 159 new enterprises were sent another mail, but only 30 (9.4%) responded to the survey. Therefore, this is a limited view of the result as it is not a representative opinion of EWs. Second, respondents to the questionnaire targeted one representative of each occupation of the SI and one representative each of the enterprise. The answers to the questionnaire items are likely to reflect the subjective opinions of the respondents, and the opinions of the workers at the workplace are unlikely to be reflected.

The strength of this study is that first, 66.4% of the SIs in Korea responded and this was thus a representative sample. It was not limited to a specific region but was targeted to institutions located in the whole country. The surveyed institutions were providing HES to enterprises of various types and sizes. Second, based on the evaluation items of the SI, opinions were surveyed using the same questionnaire in SIs and enterprises. Based on the institutional evaluation items to be implemented in the future, the contents of the HES provided at actual workplaces were compared and analyzed in detail. Therefore, these results can be used as a reference for future evaluation of SIs.

## Conclusions

It is necessary to educate EWs about the necessity of physicians to perform tasks, such as wearing a PPE and instructions to improve WE. As for nurse’s tasks, such as education about the CS and the noise work, educating the SI nurses is considered necessary as the demand for the EWs is considerable but the workload of the nurse should also be considered. As for the unique tasks of IH, such as WE management and providing instructions for wearing PPE among the tasks of IHs, training should be provided to improve the recognition of IHs’ importance given the considerable demands of EWs.

There are varieties of occupations performing health care entrusted services. Therefore, it is important to standardize the guidelines for each occupation. The standardized guidelines of the SI are developed in 2016 and based on this guidelines, work tasks should be performed for each occupation type. Assessment of SI through the standardized guidelines is also important.

In this study, the questionnaire survey was conducted for both SIs and the EWs. However, the response rate of enterprise was relatively low, and it is difficult to represent opinions of the EWs. Therefore, additional representative surveys, including EWs and workers, are required to investigate healthcare services and roles of various health occupations.

## Appendix

**Table 4:** Categories of Industry

Category	Detailed industry
Manufacturing1	
	Mining of coal, crude petroleum and natural gas, Mining of metal ores, Mining of non-metallic minerals, Manufacture of textiles, Manufacture of wearing apparel, clothing accessories and fur articles, Manufacture of wood and of products of wood and cork, Manufacture of pulp, paper and paper products, Manufacture of coke, briquettes and refined petroleum products, Manufacture of chemicals and chemical products, Manufacture of pharmaceuticals, medicinal chemical and botanical products, Manufacture of rubber and plastics products, Manufacture of basic metals, Manufacture of fabricated metal product, Manufacture of electronic components, computer; visual, sounding and communication equipment, Manufacture of medical, precision and optical instruments, watches and clocks, Manufacture of electrical equipment, Manufacture of other machinery and equipment, Manufacture of motor vehicles, trailers and semitrailers, Manufacture of other transport equipment, Manufacture of furniture
Manufacturing2	
	Agriculture, Forestry, Fishing and aquaculture, Manufacture of food products, Manufacture of beverages, Printing and reproduction of recorded media
Service business	
	Mining support service activities, Other manufacturing, Electricity, gas, steam and air conditioning supply, Water supply, Sewage, wastewater, human and animal waste treatment services, Waste collection, treatment and disposal activities; materials recovery, Remediation activities and other waste management services, Sale of motor vehicles and parts, Wholesale trade on own account or on a fee or contract basis, Retail trade, Water transport, Air transport, Warehousing and support activities for transportation, Accommodation, Publishing activities, Motion picture, video and television programme production, sound recording and music publishing activities, Broadcasting activities, Postal activities and telecommunications, Computer programming, consultancy and related activities, Information service activities, Financial service activities, except insurance and pension funding, Insurance and pension funding, Activities auxiliary to financial service and insurance activities, Real estate activities, Rental and leasing activities, Research and development, Professional services, Architectural, engineering and other scientific technical services, Other professional, scientific and technical services, Business support services, Public administration and defence; compulsory social security, Education, Human health activities, Social work activities, Creative, arts and recreation related services, Sports activities and amusement activities, Membership organizations, Repair services, Other personal services activities, Undifferentiated goods-and services-producing activities of private households for own use
Construction	
	General construction, Specialized construction activities

**Table 5:** General characteristics of specialized health management institution survey

(*N* = 81)				
Item	Classification	n(%)	no. of EW† (mean ± S.D.)	no. of person (mean ± S.D.) n(%)
Institution type				
	University hospital	19(23.5)		
	Clinic	42(51.9)		
	Corporation	20(24.7)		
Enterprise size (no. of employees)				
	50–99		83.1 ± 63.9	361,629(42.3)
	100~ 199		32.4 ± 26.6	281,588(32.9)
	200~ 299		6.3 ± 7.1	99,517(11.6)
	≥300		3.7 ± 4.0	113,084(13.2)
Categories of enterprise				
	Manufacturing1		60.6 ± 65.9	484,944(56.7)
	Manufacturing2		10.1 ± 19.5	77,690(9.1)
	Service business		34.5 ± 52.9	290,088(33.9)
	Construction		0.2 ± 0.6	3096(0.4)
Qualification of doctors				
	Specialist of occupational and environmental medicine	56(69.1)		1.5 ± 0.7
	Resident of occupational and environmental medicine	9(11.1)		1.0 ± 0.0
	Specialist of preventive medicine	21(25.9)		1.2 ± 0.5
	Doctor who had experience working in occupational health affairs	14(17.3)		1.4 ± 0.5
	Total	77(95.1)		1.9 ± 1.0
Nurse		78(96.3)		3.9 ± 2.3
Qualification of IH‡				
	Occupational health instructor	2(2.5)		1.0 ± 0.0
	professional engineers in industrial hygiene management	7(8.6)		1.0 ± 0.0
	Industrial hygiene managers	74(91.4)		2.1 ± 1.2
	Industrial hygiene control industrial engineers	31(38.3)		1.2 ± 0.5
	Total	79(97.5)		2.6 ± 1.2

†EW: entrustment workplace, ‡IH: industrial hygienist

**Table 6:** General characteristics of entrustment workplace

Items	Classification	n(%)
Categories of enterprise		
	Manufacturing1	7(23.3)
	Manufacturing2	7(23.3)
	Service business	11(36.7)
	Construction	5(16.7)
Enterprise size(no. of employees)		
	50–99	6(20.0)
	100~ 199	5(16.7)
	200~ 299	10(33.3)
	≥300	9(30.0)

## Abbreviations

95% CI: 95% Confidence interval
CS: Confined space
EW: Entrustment workplace
HCP: Hearing conservation program
HE: Health examination
IH: Industrial hygienist
OR: Odds ratio
PPE: Personal protective equipment
SI: specialized health management institution;
WE: Work environment
WTI: Work through inspection
